# Oxidative stress facilitates infection of the unicellular alga *Haematococcus pluvialis* by the fungus *Paraphysoderma sedebokerense*

**DOI:** 10.1186/s13068-022-02140-y

**Published:** 2022-05-20

**Authors:** Hailong Yan, Haiyan Ma, Yanhua Li, Liang Zhao, Juan Lin, Qikun Jia, Qiang Hu, Danxiang Han

**Affiliations:** 1 Center for Microalgal Biotechnology and Biofuels, Institute of Hydrobiology, Chinese Academy of Sciences, Wuhan, 430072 China; 2 Institute for Advanced Study, Shenzhen University, Shenzhen, 518060 China; 3 Laboratory for Marine Biology and Biotechnology, Qingdao National Laboratory for Marine Science and Technology, Qingdao, 266071 China; 4 State Key Laboratory of Freshwater Ecology and Biotechnology, Institute of Hydrobiology, Chinese Academy of Sciences, Wuhan, 430072 China; 5 College of Life Sciences, University of Chinese Academy of Sciences, Beijing, 100049 China; 6 Demeter Bio-Tech CO., LTD, Zhuhai, 519000 China; 7 Poyang Lake Eco-Economy Research Center, Jiujiang University, Jiujiang, 332005 China

**Keywords:** *Haematococcus pluvialis*, Fungal pathogen, Secondary metabolites, Oxidative stress, Antioxidant

## Abstract

**Background:**

The green microalga *Haematococcus pluvialis* is used as a cell factory for producing astaxanthin, the high-value carotenoid with multiple biological functions. However, *H. pluvialis* is prone to the infection by a parasitic fungus *Paraphysoderma sedebokerense*, which is the most devastating threat to the mass culture of *H. pluvialis* all over the world. Through dissecting the mechanisms underlying the infection process, effective measures could be developed to mitigate the pathogen threatening for the natural astaxanthin industry. By far, understanding about the interaction between the algal host and fungal pathogen remains very limited.

**Results:**

We observed that there were heat-stable substances with small molecular weight produced during the infection process and enhanced the susceptibility of *H. pluvialis* cells to the pathogen. The infection ratio increased from 10.2% (for the algal cells treated with the BG11 medium as the control) to 52.9% (for the algal cells treated with supernatant contained such substances) on the second day post-infection, indicating the yet unknown substances in the supernatant stimulated the parasitism process. Systematic approaches including multi-omics, biochemical and imaging analysis were deployed to uncover the identity of the metabolites and the underlying mechanisms. Two metabolites, 3-hydroxyanthranilic acid and hordenine were identified and proved to stimulate the infection via driving oxidative stress to the algal cells. These metabolites generated hydroxyl radicals to disrupt the subcellular components of the algal cells and to make the algal cells more susceptible to the infection. Based on these findings, a biosafe and environment-friendly antioxidant butylated hydroxyanisole (BHA) was selected to inhibit the fungal infection, which completely abolished the infection at 12 ppm. By applying 7 ppm BHA every 2 days to the algal cell culture infected with *P. sedebokerense* in the 100 L open raceway ponds, the biomass of *H. pluvialis* reached 0.448 g/L, which was comparable to that of the control (0.473 g/L).

**Conclusions:**

This study provides for the first time, a framework to dissect the functions of secondary metabolites in the interaction between the unicellular alga *H. pluvialis* and its fungal parasite, indicating that oxidative degradation is a strategy used for the fungal infest. Eliminating the oxidative burst through adding antioxidant BHA could be an effective measure to reduce parasitic infection in *H. pluvialis* mass culture.

**Graphical Abstract:**

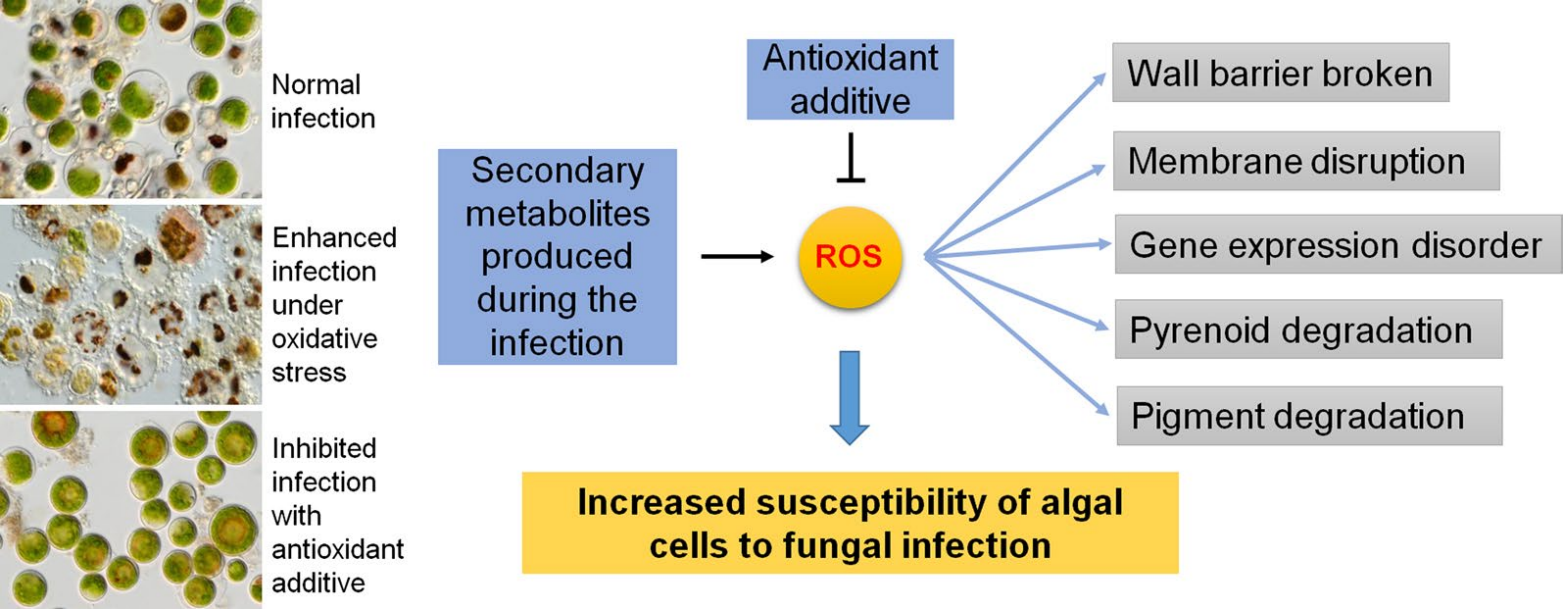

**Supplementary Information:**

The online version contains supplementary material available at 10.1186/s13068-022-02140-y.

## Background

Microalgae are increasingly used for producing high-value compounds for food, nutraceuticals and cosmetics applications and are promising resources for biofuels production [3, 48]. Microalgae also possess great potential in greenhouse gas emission mitigation as well as for wastewater treatment [6, 31, 44]. However, contaminations of parasites in mass culturing system keep threatening the sustainable production of microalgae and microalgal derived bioproducts [5]. Consequently, it is difficult to produce bulk volume of microalgal biomass at low cost due to the occurrence of various predators and pathogens in mass cultivation.

The green unicellular microalga *Haematococcus pluvialis* is a freshwater biflagellate and belongs to the class Chlorophyceae, order Volvocales, which is well known for its ability in accumulating up to 5% of the dry weight biomass of natural bio-active compound astaxanthin under stress conditions, such as nitrogen deficiency and high light irradiation [7, 15, 16, 36]. Therefore, *H. pluvialis* is considered as the most sustainable feedstock for the commercial production of astaxanthin [22], which has an estimated market value of USD 240 million in 2021 [36]. However, the development of *H. pluvialis* mass culture industry has also been retarded by contaminations of fungal parasites and other pollutants, which is often accompanied by reduced biomass yield and astaxanthin productivity [8, 43]. A parasitic fungus *Paraphysoderma sedebokerense* (Blastocladiomycota) infects the *H. pluvialis* cells in a highly species-specific manner, which is the most devastating threat to the mass culture of *H. pluvialis* all over the world [8, 18, 19]. Once the pathogen appears in the mass culturing system, the infected algal cells are dying within a very short period of time, causing severe economic loss for the natural astaxanthin manufacturing industry [12, 46]. Several strategies have been developed to control the pathogenic fungus, such as maintaining the pH of culture system at the acidic condition and application of sodium dodecylbenzene sulfonate (SDBS) [8, 19]. However, control of fungal infection by adjusting pH is not economically feasible in mass cultivation of *H. pluvialis*. In terms of surfactant SDBS, although SDBS was not detectable in the *H. pluvialis* cells, numerous studies have suggested that SDBS can cause severe environmental problems as it exhibits toxic effects on aquatic living organisms along with human cells [33, 34, 55]. More environment-friendly and biosafe measures are desired and necessary to improve the sustainability of *H. pluvialis* mass culture.

Understanding about the mechanisms underlying the infection process is essential for developing effective measures to mitigate the pathogen threatening for the natural astaxanthin industry. Interactions between *H. pluvialis* and *P. sedebokerense* have been investigated in previous studies. Sugar moiety on the *H. pluvialis* cell wall was proposed to play a role as binding molecule for recognition by *P. sedebokerense* [11]. Heat-stable recognition sites on the algal cell were necessary for the fungal attachment and encystment, while signal transduction in *H. pluvialis* cells was obligated for the fungal sporangium development and the fungal epidemic disease [2]. Interaction between the cell walls of *H. pluvialis* and carbohydrate activated enzymes in *P. sedebokerense* was believed to be essential for the parasitism process [28]. These studies focus on the specific recognition of *H. pluvialis* cells by *P. sedebokerense* and the warfare between them during the early stage of infection, but little is known about the mechanisms underlying the post-infection process. Particularly, the fast material degradation in algal cells is of great essential for developing effective measures to mitigate the pathogen threatening for the natural astaxanthin industry.

In this study, we observed that the accumulated secondary metabolites in the infection system significantly stimulated the parasitism process and enhanced the susceptibility of the algal cells to the pathogen. With the aim to uncover the identity of these metabolites and to understand the underlying mechanisms, systematic approaches including multi-omics, biochemical and imaging analysis were deployed herein. Two metabolites, i.e., 3-hydroxyanthranilic acid (3-HAA) and hordenine, were identified and proved to stimulate the infection process via causing oxidative stress to the algal cells. The hydroxyl radicals produced from these metabolites increased susceptibility of algal cells to the fungal infection by impairing the algal cell structures along with degradation of the intracellular components, and thus promoted the infection. Intentionally, application of the antioxidant butylated hydroxyanisole (BHA) to the algal–fungal system reduced the infection ratio effectively.  It indicated that oxidative degradation is a strategy used by the fungus to successfully infect algal cells, and eliminating the oxidative stress was practicable for mitigating fungal infection in *H. pluvialis* mass culture. This study provided a framework to dissect the functions of secondary metabolites in the interaction between the unicellular algal and its fungal pathogen, and developed a new crop protection measure to improve the sustainability of algal mass cultivation.

## Results and discussion

### Fungal infection caused cell death of *H. pluvialis* and the supernatant post-infection (SPI) enhanced the infection process

**Fig. 1:**
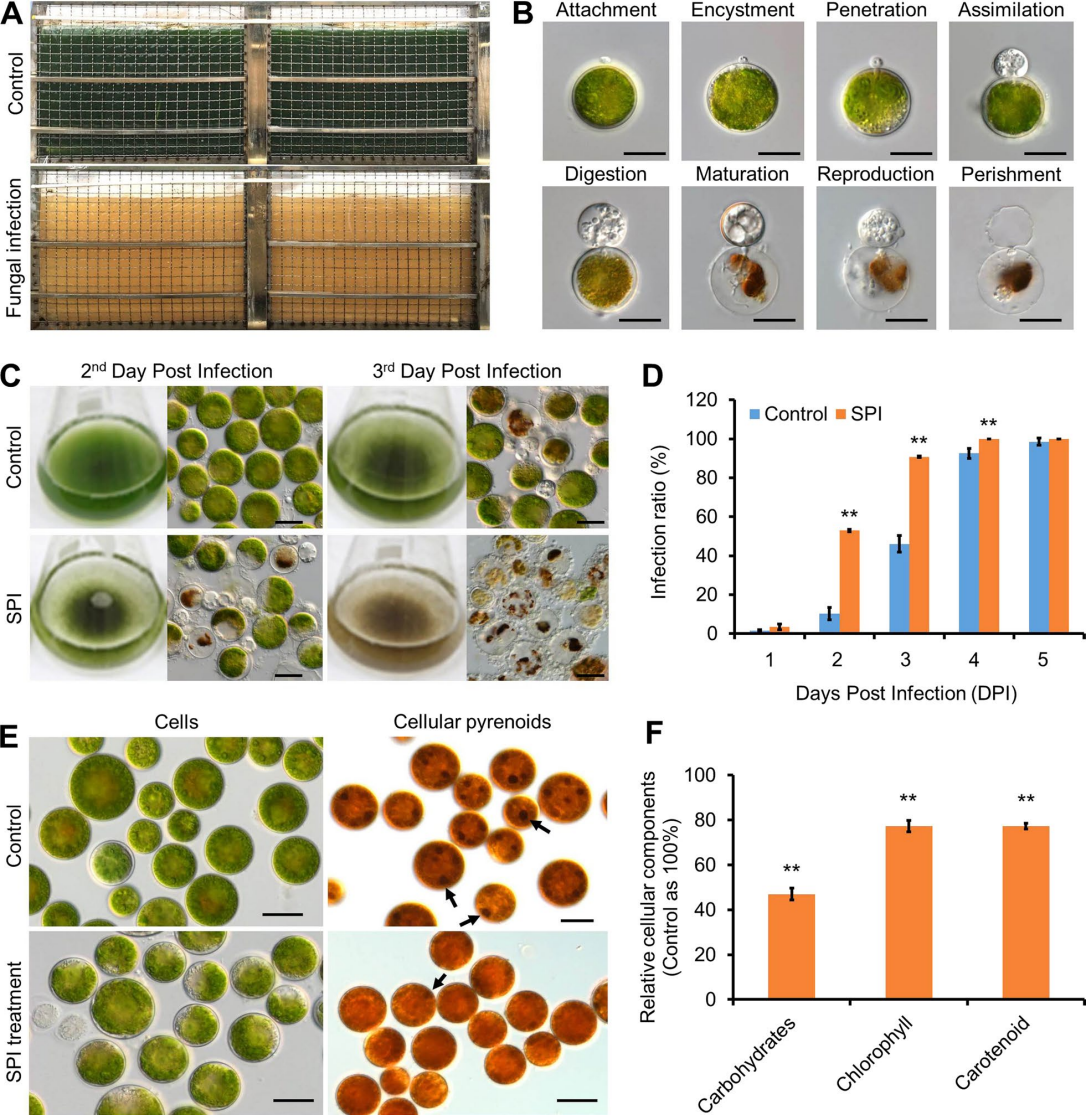
Fungal contamination crashed the *H. pluvialis* cell culture and the supernatant post-infection (SPI) enhanced the fungal infection process. **A** Crash of outdoor 360 L *H. pluvialis* algal culture after been contaminated by the fungal parasite *P. sedebokerense.* **B** Parasitic process of fungus on the algal cells. **C** SPI-treatment enhanced the fungal infection on the algal cells. **D** Infection ratio of *P. sedebokerense* on the *H. pluvialis* cells treated by SPI. **E** Morphological changes of algal cells treated with SPI. The pyrenoids were stained with the Lugol’s reagent (arrows point). **F** Changes in the algal cellular components after SPI treatment. The contents of the pigments and carbohydrates of the algal cells treated with SPI were normalized to that of the control, i.e., the algal cells treated with the BG11 medium. The quantitative data were presented as mean ± S.D. (*n* = 3). **, *p* < 0.01 (Student’s *t* test). Scale bar, 20 μm

When the *H. pluvialis* cells cultivated in outdoor 360 L panel photobioreactors were infected by *P. sedebokerense*, the algal cells died rapidly and the culture color turned from green to pale brown, which suggesting outbreak of the infection (Fig. 1A). The infection was initiated from a fungal swarmer attaching to the outside cell wall of *H. pluvialis*, followed by fungal cells encysting and degrading algal cellular components (Fig. 1B). The algal cells were rapidly consumed by the parasite to reproduce new generations (Fig. 1B). We collected the supernatant post-infection (SPI), which was prepared by removing both the host and fungal cells via centrifugation, filtration and heating in the 95 °C water bath for 15 min, to investigate the effects of SPI on the new infection process. In detail, 100 mL of algal cells (about 3.0 × 10^5^ algal cells mL^−1^) were pelleted and treated with SPI or BG11 medium, respectively, for 48 h, and then the cells were pelleted again. The treated cells were re-suspended in 100 mL of BG11 medium and challenged with 1% (*v/v*) of fungal spores (the final concentration of OD_600_ = 0.03). On the second day post-infection (DPI), the color of the control cell culture (BG11 medium-treated algal cells) was dark-green and a few algal cells were attached with the fungal swarmers. By contrast, a large number of dead and settled algal cells were observed in the culture of SPI-treated algal cells. On the third DPI, the color of the SPI-treated algal culture turned to brownish, whereas the control remained green (Fig. 1C). On the 2nd DPI, 10.2% of the algal cells were infected by the fungi in the control (i.e., those treated with the BG11 medium), whereas the infection ratio increased to 52.9% when the algal cells were treated with SPI (Fig. 1D). It was further observed that in the SPI-treated algal cells, the pigments were partially degraded and the algal cells turned translucency within 48 h (Fig. 1E). Moreover, the cellular pyrenoids were stained as dark brown with Lugol's solution in the control cells (Fig. 1E, arrows), but almost vanished in the SPI-treated cells (Fig. 1E). These results showed that the SPI degraded the algal cell components, such as pigments and pyrenoids. Quantitative analysis revealed that when compared to the control algal cells (i.e., treated with the BG11 medium), the contents of the total cellular carbohydrates and pigments (i.e., carotenoids and chlorophyll) were reduced in the algal cells treated with SPI by 50% and 20%, respectively (Fig. 1F). The SPI kept its infection-enhancing activity after heating at 95 °C for 15 min, which indicated that the activity of SPI was not mediated by enzymes. In addition, after filtered with 3000 Da cutoff membrane, the degradative activities of the SPI on carbohydrates and pigments were not significantly altered (Additional file 1: Table S1), suggesting that the activities in the SPI was most likely attributable to small molecules. These results together suggested that given heat-stable substances with small molecular weight were produced during the infection process and were capable of enhancing the susceptibility of *H. pluvialis* cells to the pathogen by affecting the algal cell integrity.

### SPI induced oxidative stresses within algal cells

**Fig. 2:**
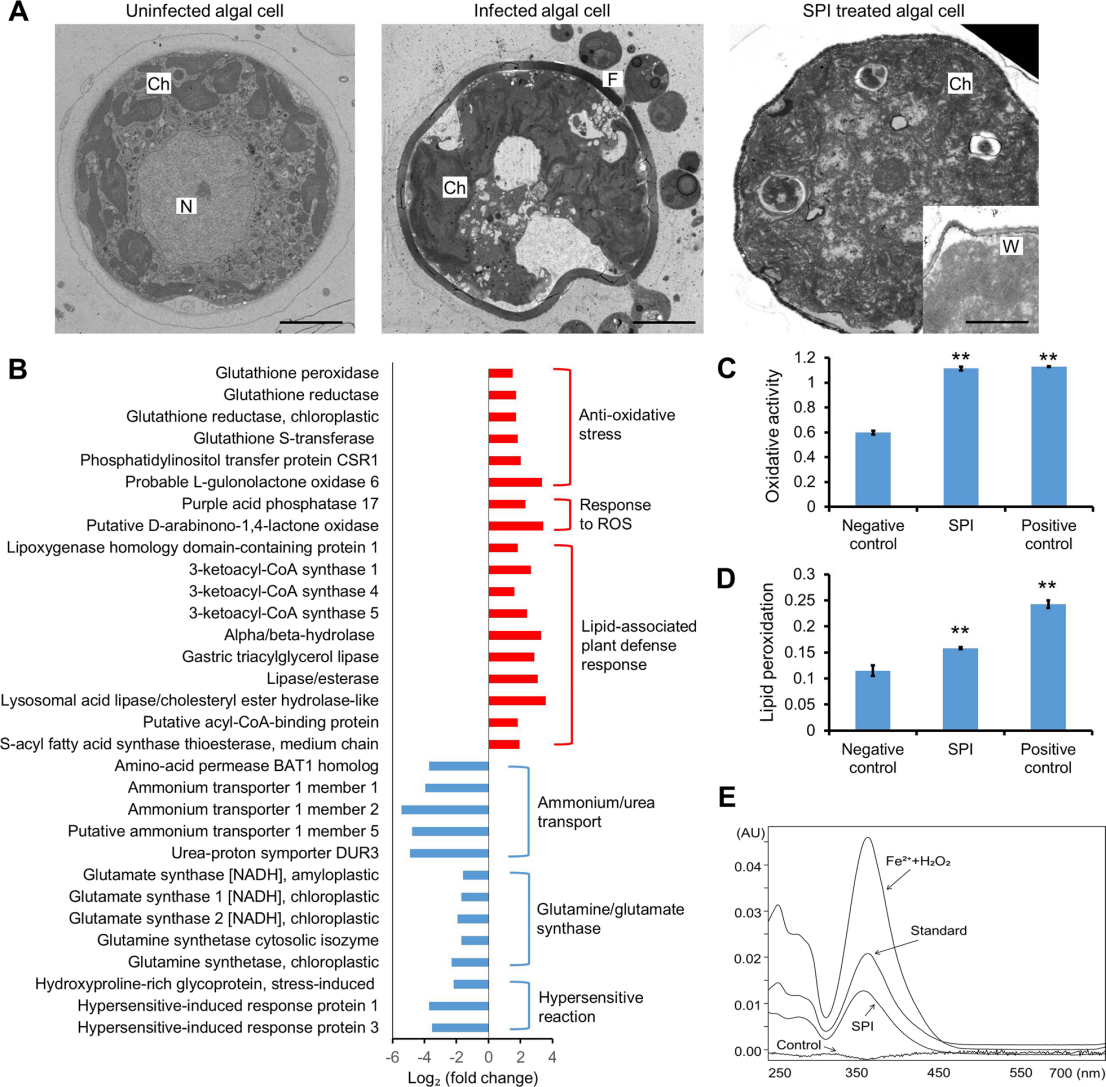
SPI caused algal structure degradation and exhibited oxidative activities both in vivo and in vitro. **A** TEM observation of the algal cells with different treatment. N, nuclear. W, cell wall. Ch, chloroplast. F, fungal cell. **B** Subset of the annotated and differentially expressed genes following SPI treatment. **C** Oxidative activities of the SPI determined by thiobarbituric acid (TBA) assay. **D** Lipid peroxidation in algal cells treated with SPI. **E** Hydroxyl radical detection. For the biochemical assays, the Fenton reagents (0.83 mM ferrous ions and 30 mM hydrogen peroxide) and BG11 medium was used as positive and negative control, respectively. The quantitative data were presented as mean ± S.D. (n = 3). **, *p* < 0.01 (Student’s *t* test). Scale bar, 5 μm

Alterations in the subcellular structures of the algal cells challenged with both *P. sedebokerense* and SPI were observed with transmission electron microscopy (TEM) (Fig. 2A). In the uninfected algal cell, a dense nucleus was located in the central of the cell and was surrounded with a cup-shaped chloroplast. The membrane structures of the subcellular organelles were distinguishable (Fig. 2A). Once being infected by the fungus, the boundaries of the nucleus of the algal cells were incomplete, and the nucleolus became fractionated. The matrix of both cytoplasm and nucleoplasm lost their homogeneity and the intact membranes were barely seen (Fig. 2A). The degradation of the cellular membranes and loosening of the cell walls were observed in the algal cells treated with SPI, indicating that SPI induced cell degradation (Fig. 2A). These results indicated that the substances in SPI degraded the algal cellular components and destructed cellular membranes and cell walls.

To facilitate understanding the effect of SPI on the algal cells, transcriptomic analysis was conducted on the algal cells treated with SPI for 24 h. A total of 11, 656 genes were annotated in the transcription, among which 259 and 146 genes were up- and down-regulated in *H. pluvialis*, respectively (Additional file 2). Since we speculated that the degradation activities of SPI might be exerted by given small molecules that can cause oxidative reactions, expression of the genes involved in oxidative stress responses were targeted and analyzed. Accordingly, it was found that expression of many genes coding for the anti-oxidative enzymes were significantly up-regulated, while genes coding for synthesis and transportation were down-regulated. Up-regulation of the genes involved in oxidative stress responses verify the hypothesis that SPI may contain substances that can cause the generation of reactive oxygen species (ROS) [17, 49].

To further test the hypothesis, the oxidative activities of SPI were measured using the thiobarbituric acid (TBA) assay with Fenton reagent as the positive control, because it is a known reaction that generates oxidative stress through small molecules [1, 9]. The results showed that the SPI possessed strong oxidative activity in vitro (Fig. 2C). In addition, the SPI showed lipid peroxidation activity when acting on the algal cellular membranes, leading to formation of malondialdehyde in vivo (Fig. 2D). To identify the ROS produced by SPI, the dimethyl sulfoxide trapping method was used and the results suggested that SPI produced hydroxyl radical in vitro (Fig. 2E).

Based on the transcriptomic results and a suite of observations and biochemical assays, it can be concluded that SPI contained substances that exerted oxidative stresses via generation of ROS in the algal cells. Oxidative degradation of the algal subcellular structures might be the cause of decreased resistance to fungal infection.

### Secondary metabolites mediated Fenton reaction facilitated the fungal infection

**Fig. 3:**
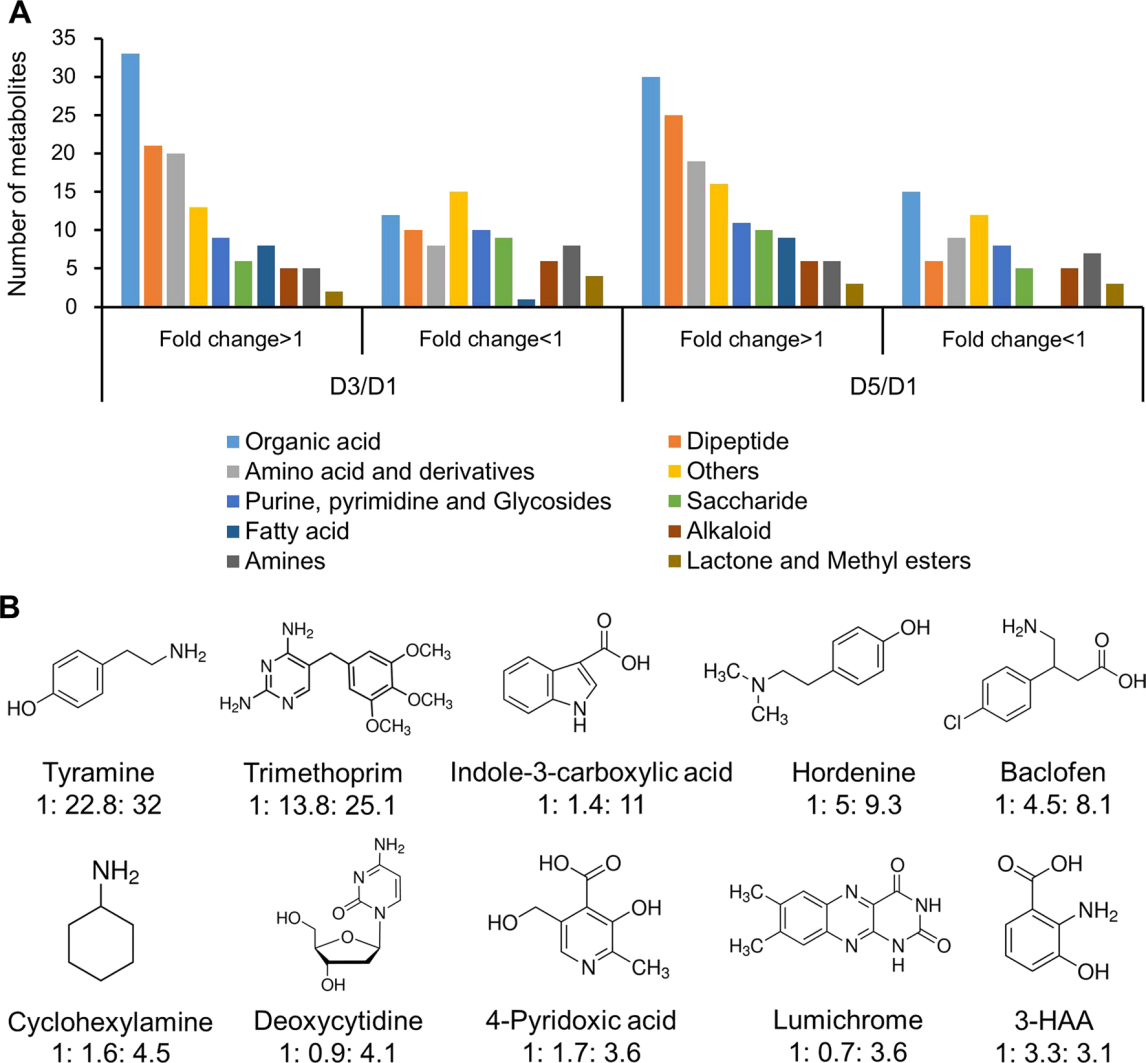
Identification of the metabolites with putative functions in causing oxidative stresses in the algal cells through comparative metabolomics analysis. **A** Relative fold changes (FC) and the number of annotated metabolites in the SPIs collected on 1, 3 and 5 day post-infection. **B** Ten metabolites (D5/D1 FC > 2) with putative functions involved in driving Fenton reaction were screened

Metabolomic analysis was performed to identify the small molecules causing the oxidative stresses. The SPIs were collected at different infection stages, i.e., 1, 3 and 5 day post-inoculation of the fungus into the algal cell cultures. The degradation activity of SPI was represented by the pigment degradation ratio. It was found that the degradation activity of SPI collected on day (D) 5 was significantly higher than that on D1 (Additional file 1: Fig. S1), suggesting that the concentration of the target metabolites increased over 5 days. Based on this, 62 metabolites, which showed over twofold increase on D5 than that on D1, were selected from the metabolomic data (Additional file 3). Most of these metabolites were organic acids, dipeptides, amino acids and derivatives (Fig. 3A). Ten metabolites, including tyramine, trimethoprim, indole-3-carboxylic acid, hordenine, deoxycytidine, 4-pyridoxic acid, lumichrome, 3-hydroxyanthranilic acid (3-HAA), baclofen and cyclohexylamine, were retrieved as they contain the chemical moiety of phenol/quinone/aromatic (Fig. 3B). Such types of compounds are known to be able to mediate the Fenton reaction and degrade biomolecules by producing hydroxyl radicals [23].

**Fig. 4:**
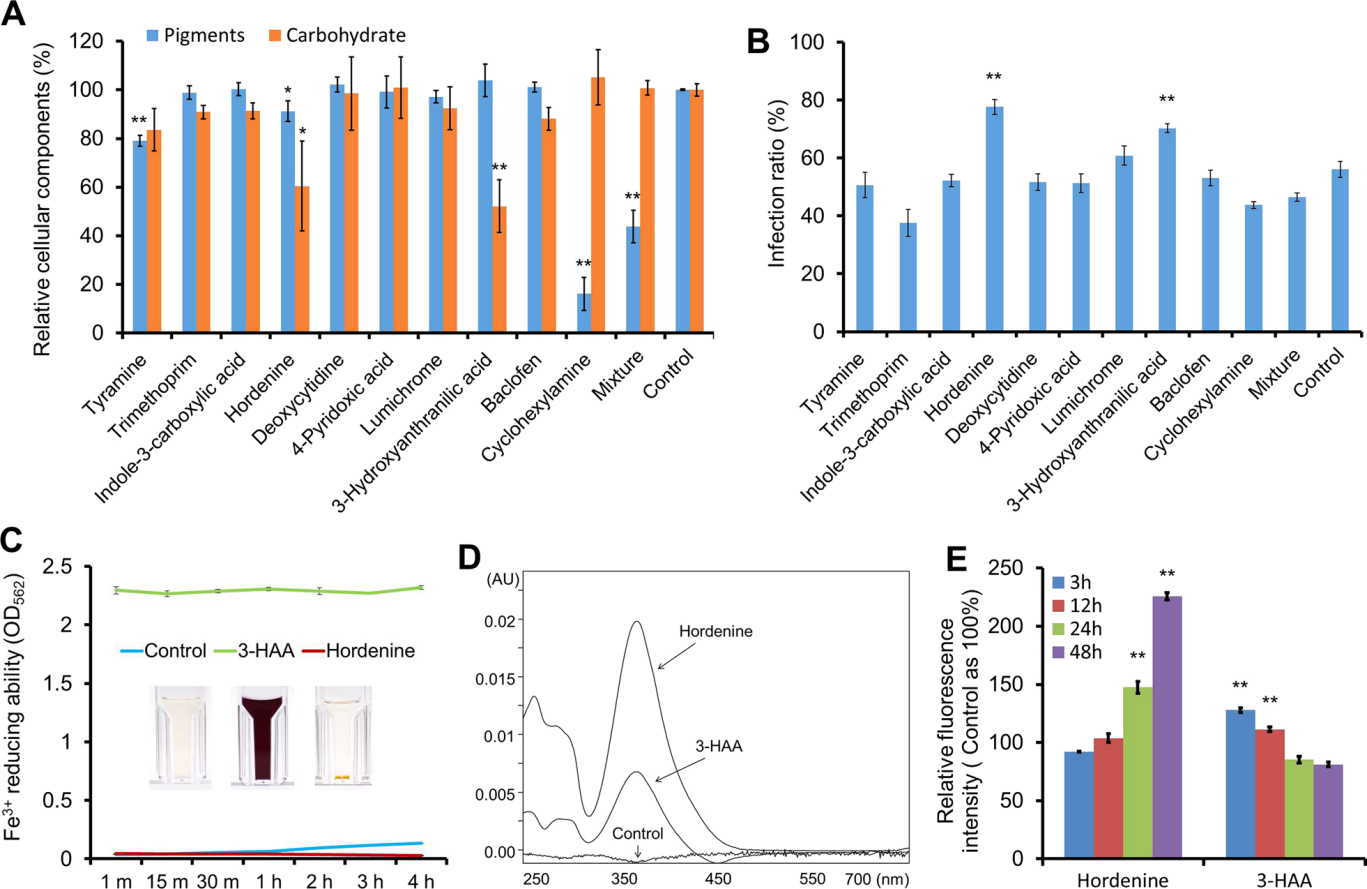
Effects of the screened metabolites on the algal cellular composition and infection process. **A** Degradation of the algal cellular components by the screened metabolites. The control and the mixture was the algal cells treated with BG11 medium and 10 reagents that mixed in equal volume, respectively. **B** Effects of the screened metabolites on the infection ratio. **C** Fe^3+^ reducing ability of 3-HAA and hordenine. **D** Production of hydroxyl radicals by hordenine and 3-HAA. **E** Relative intracellular ROS intensity of algal cells after treated with hordenine or 3-HAA, determined by DCFH-DA staining. The fluorescence intensity of the BG11 medium-treated algal cells (control) was considered as 100%. The concentration of cyclohexylamine was 2% (*v*/*v*), and the concentration of the other 9 reagents were 0.2% (*w*/*v*) in the assays. The solutions were prepared with the BG11 medium and heated in the 95 °C water bath for 15 min, followed by ultrasonic treatment for 5 min and filtrated with 3000 Da cut off membrane prior to use. The quantitative data were presented as mean ± S.D. (n = 3). *, *p* < 0.05, **, *p* < 0.01 (Student’s *t* test). The Fe^3+^ reducing ability and hydroxyl radical productivity of the other 8 metabolites can be referred to Figures S3 and S4 (Additional file 1)

When these substances were added into the algal cell culture, it was first observed that hordenine and 3-HAA significantly reduced the contents of carbohydrates, while tyramine, hordenine and cyclohexylamine caused degradation of the pigments in the treated algal cells (Fig. 4A). By microscopic observation, it was found that the three metabolites impaired the pigment in different manners. Tyramine and hordenine degraded the intracellular pigments, whereas cyclohexylamine disrupted the cell membrane and caused the effluxion of pigments (Additional file 1: Fig. S2). Secondly, the potential infection-prompting effect of the candidate metabolites was checked. On the 3^rd^ DPI, the algal cells treated with either hordenine or 3-HAA showed significantly enhanced infection ratio than that of the control (i.e., the algal cells treated with BG11 medium) and other compounds (Fig. 4B). The Fenton reaction is initiated from the reduction of Fe^3+^ to Fe^2+^, which is the key factor for driving Fenton reaction [21]. Thus the reducing activities of the 10 candidate metabolites were tested. Among them, 3-HAA and lumichrome showed strong activity in reducing Fe^3+^ to Fe^2+^ (Fig. 4C, Additional file 1: Fig. S3). In addition, 3-HAA and hordenine generated hydroxyl radical in the assay with DMSO as substrate, but no hydroxyl radical productivity was detected with the other 8 metabolites (Fig. 4D, Additional file 1: Fig. S4). Based on these experiments, 3-HAA and hordenine were considered as the molecules that could cause oxidative stresses in vitro. To verify their functions, 3-HAA and hordenine was added to the algal cell culture, respectively, and the intracellular concentration of hydrogen peroxide (H_2_O_2_), an important intermediate of the Fenton reaction, was measured by staining the algal cells with the fluorescence dye 2’, 7’-dichlorodihydrofluorescein diacetate (DCFH-DA). The relative fluorescence intensity was detected with a flow cytometer, and the algal cells treated in BG11 medium were used as the control. The result showed that in the hordenine-treating algal cells the relative concentration of H_2_O_2_ continued increasing during 48 h, while the level of H_2_O_2_ in the 3-HAA-treating algal cells transiently increased during 24 h and then gradually decreased (Fig. 4E).

These results taken together indicated 3-HAA and hordenine were the possible components in SPI causing oxidative stresses in the algal cells through mediating Fenton reaction, as 3-HAA can reduce Fe^3+^ to Fe^2+^ and they both produce H_2_O_2_, which are the two substrates of Fenton reaction. The metabolite 3-HAA is an intermediate of kynurenine pathway widely found in bacteria, yeast, fungi, plants and mammals, and it has been reported to be a fungal producing mediator that was widely applied in Fenton processes in dye decolorization due to its ability in reducing Fe^3+^ to Fe^2+^ [41, 42]. It is also a generator of free radicals through its auto-oxidation [4, 26]. Thus, the generation of H_2_O_2_ from the auto-oxidation of 3-HAA could not be excluded here. Hordenine, originally detected in barley and also isolated from marine alga *Phyllophora nervosa* [13, 32], is a phenethylamine alkaloid with various bioactivities, including antibiotic activity against microorganisms, and inhibition of quorum sensing and biofilm formation [35, 56]. It was reported that hordenine was responsible for the protective responses of plants to various stresses through jasmonate dependent defense pathway [20], and also acted as a plant allelochemical that can inhibit the growth of weed or defend against pathogens attack [24, 25].

### Application of antioxidant to inhibit the fungal infection

**Fig. 5:**
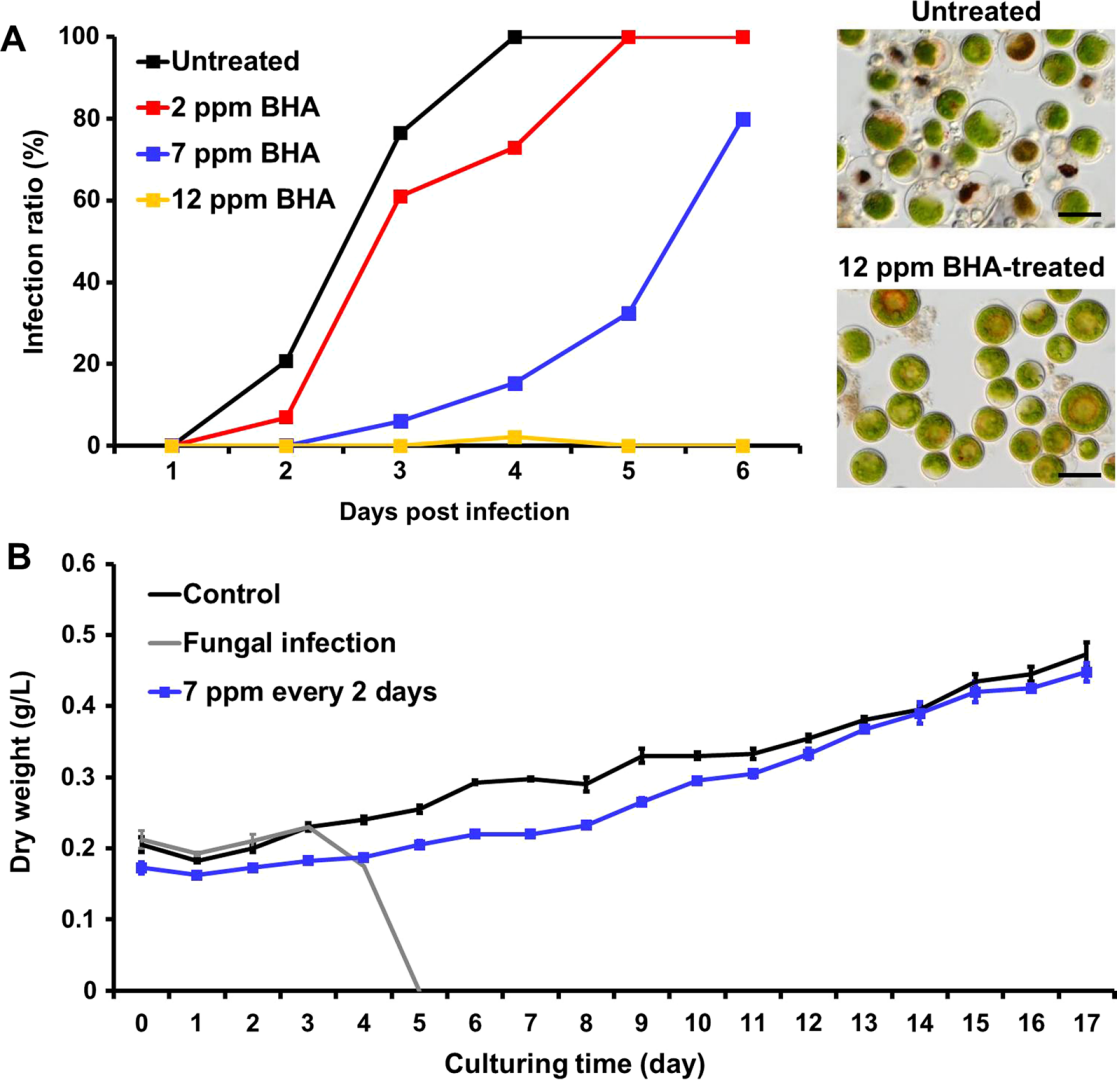
Inhibitory effect of BHA on the fungal infection. **A** Infection process was inhibited by the addition of BHA at various concentrations. Microscopic images showed the untreated and 12 ppm BHA-treated cell cultures on the 3^rd^ day post-infection, respectively. **B** Dry weight of *H. pluvialis* cell culture in the 100 L open raceway pond was rescued by applying 7 ppm BHA every 2 days. Control, the uninfected and untreated cell culture. Fungal infection, the infected  and untreated cell culture. 7 ppm every 2 days, the infected cell culture treated with 7 ppm BHA every 2 days. Scale bar, 20 μm

As the oxidative stress caused by the SPI impaired the algal cell structures and promoted the fungal infection process, an exogenous antioxidant was introduced to relieve such oxidative stress in the culture to inhibit the infection. BHA is one of the most commonly used synthetic antioxidants in food and biodiesel fuels to prevent oxidation for its low cost, high stability and effectiveness [37, 39, 40, 54]. In addition, BHA is biosafe and environmental-friendly, which rendering its application in the aquaculture industry [38, 53]. BHA was added into the infection system at different final concentrations (i.e., 2 ppm, 7 ppm and 12 ppm) (Fig. 5A). The infection ratio of the newly infected algal cells was calculated to reflect the infection inhibitory effect. Compared to the untreated *H. pluvialis* culture, addition of BHA at 2 ppm delayed the complete fungal infection for 1 day. Elevating the concentration of BHA to 7 ppm decreased the infection greatly, and the infection was only about 30% on day 5, when the untreated group was 100% infected. Application of 12 ppm BHA to the system completely suppressed the fungal infection (Fig. 5A). Based on microscopic observation, it was found that 76.4% of the untreated algal cells were infected by *P. sedebokerense* on the 3^rd^ DPI, while the algal cell culture treated with 12 ppm of BHA kept healthy (less than 1% infection) (Fig. 5A). To further evaluate the inhibitory effect of BHA, it was added to the algal culture in a 100 L open raceway ponds. BHA (7 ppm) was applied every other day to the infected algal cell culture. As shown in Fig. 5B, the dry weight of the uninfected algal cell culture (control) reached 0.473 g/L after 17 day cultivation. However, the infected algal cell culture crashed quickly after 4–5 days without applying BHA, and the algal cells flocculated and sank to the bottom of the pond. By applying 7 ppm BHA every other day to the algal cell culture with fungal contamination, the infection was effectively inhibited and the final biomass yield reached 0.448 g/L on the 17th day, which was almost identical to that of the control. These data suggested that BHA was an effective chemical that blocked the fungal infection in mass culture of *H. pluvialis* (Fig. 5B).

According to all the results described above, a model was proposed herein to illustrate the major findings of this study. Production of secondary metabolites such as 3-HAA and hordenine in the infection system mediated the generation of ROS via Fenton reaction. The ROS disrupted the *H. pluvialis* cell wall barrier, impaired the algal cell membrane systems, and degraded the algal subcellular components, which made the algal cells more susceptible to the fungal infection. However, the antioxidant BHA at the extremely low concentration blocked the infection completely, indicating that the oxidative burst is essential for the pathogens to infest the host algal cells.

## Conclusions

In this study, two secondary metabolites (i.e., 3-HAA and hordenine) were identified from the fungal infection system, which drove severe oxidative stresses on the host cells to facilitate the infection of *H. pluvialis* by the fungus *P. sedebokerense*. Based on these findings, the antioxidant BHA was introduced and successfully blocked the fungal infection. This proof-of-concept study indicated that utilization of antioxidants represents a novel strategy to reduce the fungal infection in *H. pluvialis* and potentially in other microalgal mass cultures.

## Methods

### Biological material cultivation conditions

*H. pluvialis* strain K-0084 was acquired from the Scandinavian Culture Center for Algae and Protozoa at the University of Copenhagen, Denmark, and cultured in the BG11 growth medium [45] at 21–23 °C under continuous illumination (20 μmol·m^−2^·s^−1^). For outdoor 360 L cultivation of *H. pluvialis*, an initial algal cell density of 5.0 × 10^4^ mL^−1^ were inoculated in plastic films filled with BG11 medium. Daily sampling was performed to monitor the fungal infection process under microscope (Olympus, BX53 with a DP70 CCD camera). The fungal parasite *P. sedebokerense* used in this study was isolated in the previous study [28]. *P. sedebokerense* cells were grown in the fungal growth medium supplemented with yeast extract and peptone [18] on an orbital shaker at a speed of 150 rpm maintained at 30 °C.

### Preparation of the supernatant post-infection (SPI) and SPI incubation assay

When the fungal cell was cultured for   5 days, 1% (*v*/*v*) of the fungal cells (final concentration of OD_600_=0.03) was inoculated in 100 mL of the algal cell cultures (about 3.0 × 10^5^ algal cells mL^−1^) in 250 mL flasks. The mixture was incubated on an orbital shaker at a speed of 150 rpm at 30 °C. Samplings were performed on a daily basis and the infection ratios [11] were determined by cell counting under microscope. Over 1000 algal cells were examined and the infected algal cells were counted to calculate the infection ratios.

To prepare the SPI, when the infection ratio was 100%, the culture mixture was centrifuged at 2,100 g for 5 min. The supernatant was collected and filtrated with double-layered 1.2 μm glass microfiber filters (Whatman, UK). The filtrate was further heated at 95 °C in water bath for 15 min and was stored at 4 °C. For detecting the infection enhancing activity of the SPI, 100 mL of the algal cell culture (5.0 × 10^5^ cells mL^−1^) from the exponential growth phase was centrifuged at 700 g for 3 min, and the cell pellet was suspened in 100 mL SPI or BG11 medium and was incubated for 48 h at 30 °C, under light intensity of 20 μmol·m^−2^·s^−1^ at 150 rpm. The supernatant was removed via centrifugation at 700 g for 3 min, and the cell pellet was re-suspended in 100 mL of BG11 medium and was challenged with fungal spores as described above. To calculate the infection ratio, three independent biological replicates were included and the quantitative data were presented as mean ± S.D. (n = 3). *, *p* < 0.05, **, *p* < 0.01 (Student’s *t* test).

### Transmission electron microscopy and pyrenoids staining

The healthy *H. pluvialis* cells that grown till the logarithmic phase and those incubated with SPI for 3 days were centrifuged at 700 g for 3 min, respectively. The cell pellet was washed twice with the fresh BG11 medium, and was fixed overnight in 2.5% (*v/v*) glutaraldehyde at 4 °C. The following sample processing procedures included osmium fixation, dehydration, infiltration, polymerization, thin-sectioning and staining, which were refered to Wayama [51]. The fixed and stained samples were examined with transmission electron microscope (FEI, Tecnai G2 20 TWIN, 0.24 nm/200 kV). For pyrenoids staining, the algal cell samples were fixed with 2% (v/v) of the Lugol's solution and incubated for 5 min. The pyrenoids were stained as brown-colored particles under microscopic observation.

### Biochemical analysis

SPI or BG11 medium-treated algal cells were pelleted via centrifugation at 700 g for 3 min and the cell pellet was washed with distilled H_2_O. The cell pellet was ground to fine powder in a mortar on ice under dim light, and the pigments were extracted with dimethyl sulfoxide (DMSO) for several times until the cell debris turned pale. The cellular content of pigments (chlorophyll and carotenoid) was quantified using a spectrophotometer [52]. The cells were hydrolyzed by 3 M trifluoroacetic acid (TFA) at 98 °C for 4 h. The liquid was dried under nitrogen gas, and the residue was re-dissolved in distilled H_2_O. After filtered with 0.22 μm micro-aperture filter membrane (Merck Millipore, USA), the sugar content in the sample was analyzed using an ICS 5000+ Ion Chromatography (IC) system (Thermo-Fisher Scientific, USA) [28]. A 3000 Da cutoff membrane (Amicon Ultra-15, 3 kDa MWCO, Germany) was used to remove substances with large molecular weight in the SPI, and the filtrate was termed as filtered SPI and was used to treat *H. pluvialis* cells for 48 h. The content of carbohydrates and pigments of the algal cells was quantified, respectively, to reflect the degradation activity of the filtered SPI. Thiobarburic acid (TBA) assay was introduced to detect the oxidative activity of SPI [27]. Fenton reagents (0.83 mM ferrous ions and 30 mM hydrogen peroxide) and BG11 medium were used as the positive and negative control, respectively. The Malondialdehyde (MDA) kit (Jiancheng Bioengineering Institute, Nanjing, China) was introduced to measure the in vivo lipid peroxidation in the SPI-treated *H. pluvialis* cells. Ferrozine assay was conducted to measure the ferric ions reducing activity [10].

### Hydroxyl radical and hydrogen peroxide assays

The hydroxyl radicals oxidize DMSO and generate formaldehyde, which can react with 2, 4-dinitrophenylhydrazine (DNPH) and form hydrazone (HCHO–DNPH).HCHO–DNPH has maximum absorbance at 355 nm and can be detected by High Performance Liquid Chromatography (HPLC) [47]. Fenton reagent (0.2 mM Fe^2+^ and 8 mM H_2_O_2_) and BG11 medium were used as the positive and negative control, respectively. Formaldehyde was introduced as standard for the quantitatively analysis. Specifically, the given substance was mixed with 250 mM DMSO to the final volume of 2 mL. After incubating at 25 °C for 48 h, 2.5 mL of H_3_PO_4_–NaH_2_PO_4_ (pH 4.0) and 0.2 mL of 6 mM DNPH was added to the mixture, and was then diluted to the volume of 5 mL with distilled water. The mixtures were maintained at room temperature for 2 h and analyzed with HPLC (Waters alliance e2695 LC with a 2998 PDA detector, USA). The mobile phase was  methanol and water (60:40, v/v), and the flow rate was 1 mL·min^−1^. Under such conditions, the peaks were well separated within 10 min.

The intracellular hydrogen peroxide (H_2_O_2_) was determined by 2’, 7’-dichlorodihydrofluorescein diacetate (DCFH-DA, Sigma-Aldrich, USA) staining [14]. The concentration of DCFH-DA in the working solution was 10 μM. The fluorescence intensity of the stained cells was measured by a flow cytometer (Beckman Coulter, Inc. FC-500, USA), and the excitation/emission wavelength was 488/525 nm. The fluorescence intensity of DCFH-DA in the given substance-treated cells was compared to that of the BG11 medium-treated cells (as 100%) to reflect the changes of intracellular H_2_O_2_ level that caused by the metabolite.

### Metabolomics

The SPIs of 1, 3 and 5 day post-infection were collected, respectively. The substances in the sample were analyzed by ultra-high-performance liquid chromatography coupled with hybrid quadrupole time-of-flight mass spectrometry (UHPLC–QTOF–MS). Two independent biological repeats were conducted for each sample. The relative concentration of each metabolite in each sample was calculated as following:

$${\text{Peak intensity}}\,{ = }\,{\text{Metabolite }}\left( {\text{Peak Area}} \right){\text{ / Sum (Peak Area)}}$$

where Metabolite (Peak Area) is the peak area of each metabolite, Sum (Peak Area) is the sum of the peak areas of all the metabolites detected in the very sample.

Fold change (peak intensity of D5/D1) > 3 was screened and compounds with phenol/quinone/aromatic moiety were particularly targeted as such moiety may have the activity to drive Fenton reaction [23]. Target chemicals were purchased from Sigma-Aldrich, various concentrations and solvents were tested in preliminary experiments to determine the optimal way of preparing the selected chemicals presented in the results.

### RNA extraction and RNA-seq analysis

For analysis of algal response to SPI treatment, 100 mL of the *H. pluvialis* cell culture (5.0 × 10^5^ cells mL^−1^) were incubated in SPI or BG11 medium (as control) for 24 h, respectivley. One milliliter of the algal cell culture was sampled and centrifuged. The cell pellet was frozen with liquid nitrogen immediately. The RNA extraction and RNA-seq analysis were performed according to the methods described by Ma and Lin [28, 30]. Three independent biological replicates were included for RNA preparation and RNA-seq. The reference transcriptome of *H. pluvialis* was constructed using the Trinity platform (version 2.5.1) for de novo assembly without genome reference. The assembly quality and completeness were assessed by computing the E90N50 value, examining RNA-Seq Read Representation and searching orthologs against the chlorophyte sets from OrthoDB version 9 (https://busco.ezlab.org/) using the nematod BUSCO [50]. The transcriptome was functionally annotated using Trinotate (https://trinotate.github.io/), which employs a number of well-established methods for functional annotation, including homology search to known sequence data (BLAST+ and NCBI). Differentially expressed (DE) genes were identified using Bioconductor packages DESeq2 [29]. The genes of which transcripts showed more than a fourfold change (|log2 Fold change|> 2) in the SPI-treated samples compared with the control, and with a false discovery rate (FDR)-corrected *P* value < 0.01, were considered as significantly differentially expressed.

### Application of BHA to *H. pluvialis* cell culture

BHA was dissolved in ethanol to the concentration of 5 × 10^4^ ppm as stock solution. A set of 100 mL of algal cell cultures (about 3.0 × 10^5^ algal cells mL^−1^) in 250 mL flasks were inoculated with 1% (*v/v*) of fungal cells, and was supplied with BHA at various final concentrations. The algal cells were cultured on an orbital shaker at a speed of 150 rpm at 30 °C under light intensity of 20 μmol·m^−2^·s^−1^. For the scaling-up experiment, BHA (7 ppm) was applied to 100 L open raceway ponds every 2 day for 17 days. The fungus was inoculated on the first day of cultivation at the ratio of 0.1% (*v/v*). Daily sampling was performed for microscopic observation to monitor the fungal infection process.

## Supplementary Information

**Additional file 1: Table S1.** Activity of the filtrated SPI in degrading cellular carbohydrates and pigments. **Figure S1.** Pigment degrading activity of SPI harvesting from the algal cultures at various infection stage. **Figure S2.** Morphological changes of the algal cells treated with the screened metabolites. **Figure S3.** Fe^3+^ reducing activity of the screened metabolites. **Figure S4.** Hydroxyl radical production of the screened metabolites.

**Additional file 2:** Differentially expressed genes.

**Additional file 3:** Metabolomics data of SPIs harvested at various infection stage.

## Data Availability

Transcriptome data are available at NCBI Sequence Read Archive (https://www.ncbi.nlm.nih.gov/sra) with the accession number: PRJNA720251.

## References

[1] Arantes V, Jellison J, Goodell B (2012). Peculiarities of brown-rot fungi and biochemical Fenton reaction with regard to their potential as a model for bioprocessing biomass. Appl Microbiol Biotechnol.

[2] Asatryan A, Boussiba S, Zarka A (2019). Stimulation and Isolation of *Paraphysoderma sedebokerense* (Blastocladiomycota) propagules and their infection capacity toward their host under different physiological and environmental conditions. Front Cell Infect Microbiol.

[3] Borowitzka MA, Vonshak A (2017). Scaling up microalgal cultures to commercial scale. Eur J Phycol.

[4] Breton J, Avanzi N, Magagnin S, Covini N, Magistrelli G, Cozzi L, Isacchi A (2000). Functional characterization and mechanism of action of recombinant human kynurenine 3-hydroxylase. Eur J Biochem.

[5] Carney LT, Lane TW (2014). Parasites in algae mass culture. Front Microbiol.

[6] Choi HI, Hwang S-W, Sim SJ (2019). Comprehensive approach to improving life-cycle CO_2_ reduction efficiency of microalgal biorefineries: a review. Biores Technol.

[7] Damiani MC, Popovich CA, Constenla D, Leonardi PI (2010). Lipid analysis in *Haematococcus pluvialis* to assess its potential use as a biodiesel feedstock. Biores Technol.

[8] Ding Y, Zhang A, Wen X, Wang Z, Wang K, Geng Y, Li Y (2020). Application of surfactants for controlling destructive fungus contamination in mass cultivation of *Haematococcus pluvialis*. Biores Technol.

[9] Eastwood DC, Floudas D, Binder M, Majcherczyk A, Schneider P, Aerts A, Asiegbu FO, Baker SE, Barry K, Bendiksby M, Blumentritt M, Coutinho PM, Cullen D, Vries RPd, Gathman A, Goodell B, Henrissat B, Ihrmark K, Kauserud H, Kohler A, LaButti K, Lapidus A, Lavin JL, Lee Y-H, Lindquist E, Lilly W, Lucas S, Morin E, Murat C, Oguiza JA, Park J, Pisabarro AG, Riley R, Rosling A, Salamov A, Schmidt O, Schmutz J, Skrede I, Stenlid J, Wiebenga A, Xie X, Kües U, Hibbett DS, Hoffmeister D, Högberg N, Martin F, Grigoriev IV, Watkinson SC (2011). The plant cell wall-decomposing machinery underlies the functional diversity of forest fungi. Science.

[10] Gibbs CR (1976). Characterization and application of FerroZine iron reagent as a ferrous iron indicator. Anal Chem.

[11] Gutman J, Zarka A, Boussiba S (2011). Evidence for the involvement of surface carbohydrates in the recognition of *Haematococcus pluvialis* by the parasitic blastoclad *Paraphysoderma sedebokerensis*. Fungal Biol.

[12] Gutman J, Zarka A, Boussiba S (2009). The host-range of *Paraphysoderma sedebokerensis*, a chytrid that infects *Haematococcus pluvialis*. Eur J Phycol.

[13] Güven KC, Percot A, Sezik E (2010). Alkaloids in marine algae. Mar Drugs.

[14] Gwak Y, Hwang Y-S, Wang B, Kim M, Jeong J, Lee C-G, Hu Q, Han D, Jin E (2014). Comparative analyses of lipidomes and transcriptomes reveal a concerted action of multiple defensive systems against photooxidative stress in *Haematococcus pluvialis*. J Exp Bot.

[15] Han DX, Li YT, Hu Q (2013). Astaxanthin in microalgae: pathways, functions and biotechnological implications. Algae.

[16] Harker M, Tsavalos AJ, Young AJ (1996). Factors responsible for astaxanthin formation in the Chlorophyte *Haematococcus pluvialis*. Biores Technol.

[17] Hasanuzzaman M, Bhuyan MHMB, Zulfiqar F, Raza A, Mohsin SM, Mahmud JA, Fujita M, Fotopoulos V (2020). Reactive oxygen species and antioxidant defense in plants under abiotic stress: revisiting the crucial role of a universal defense regulator. Antioxidants (Basel, Switzerland).

[18] Hoffman Y, Aflalo C, Zarka A, Gutman J, James TY, Boussiba S (2008). Isolation and characterization of a novel chytrid species (phylum Blastocladiomycota), parasitic on the green alga Haematococcus. Mycol Res.

[19] Hwang S-W, Choi HI, Sim SJ (2019). Acidic cultivation of *Haematococcus pluvialis* for improved astaxanthin production in the presence of a lethal fungus. Biores Technol.

[20] Ishiai S, Kondo H, Hattori T, Mikami M, Aoki Y, Enoki S, Suzuki S (2016). Hordenine is responsible for plant defense response through jasmonate-dependent defense pathway. Physiol Mol Plant Pathol.

[21] Kameshwar AKS, Qin W (2018). Molecular networks of postia placenta involved in degradation of lignocellulosic biomass revealed from metadata analysis of open access gene expression data. Int J Biol Sci.

[22] Khoo KS, Lee SY, Ooi CW, Fu X, Miao X, Ling TC, Show PL (2019). Recent advances in biorefinery of astaxanthin from *Haematococcus pluvialis*. Biores Technol.

[23] Korripally P, Timokhin VI, Houtman CJ, Mozuch MD, Hammel KE (2013). Evidence from Serpula lacrymans that 2, 5-dimethoxyhydroquinone Is a lignocellulolytic agent of divergent brown rot basidiomycetes. Appl Environ Microbiol.

[24] Kotzamani A, Vasilakoglou I, Dhima K, Moulas AN, Vaiou M, Stefanou S (2021). Impact of soil salinity on barley allelopathic potential and main secondary metabolites gramine and hordenine. J Plant Growth Regul.

[25] Lebecque S, Crowet J-M, Lins L, Delory BM, du Jardin P, Fauconnier M-L, Deleu M (2018). Interaction between the barley allelochemical compounds gramine and hordenine and artificial lipid bilayers mimicking the plant plasma membrane. Sci Rep.

[26] Li K, Horanyi PS, Collins R, Phillips RS, Eriksson K-EL (2001). Investigation of the role of 3-hydroxyanthranilic acid in the degradation of lignin by white-rot fungus *Pycnoporus cinnabarinus*. Enzyme Microb Technol.

[27] Li X (2013). Solvent effects and improvements in the deoxyribose degradation assay for hydroxyl radical-scavenging. Food Chem.

[28] Lin J, Yan H, Zhao L, Li Y, Nahidian B, Zhu M, Hu Q, Han D (2021). Interaction between the cell walls of microalgal host and fungal carbohydrate-activate enzymes is essential for the pathogenic parasitism process. Environ Microbiol.

[29] Love MI, Huber W, Anders S (2014). Moderated estimation of fold change and dispersion for RNA-seq data with DESeq2. Genome Biol.

[30] Ma H, Wu X, Wei Z, Zhao L, Li Z, Liang Q, Zheng J, Wang Y, Li Y, Huang L, Hu Q, Han D (2020). Functional divergence of diacylglycerol acyltransferases in the unicellular green alga *Haematococcus pluvialis*. J Exp Bot.

[31] Maity JP, Bundschuh J, Chen C-Y, Bhattacharya P (2014). Microalgae for third generation biofuel production, mitigation of greenhouse gas emissions and wastewater treatment: present and future perspectives—A mini review. Energy.

[32] Mann JD, Steinhart CE, Mudd SH (1963). Alkaloids and Plant Metabolism V. The distribution and formation of tyramine methylpherase during germination of barley. J Biol Chemis.

[33] Mu Z, Liu X, Zhao Y, Zhang J (2014). Cytotoxic effects of sodium dodecyl benzene sulfonate on human keratinocytes are not associated with proinflammatory cytokines expression. Chin Med J.

[34] Qv X-Y, Jiang J-G (2013). Toxicity evaluation of two typical surfactants to *Dunaliella bardawil*, an environmentally tolerant alga. Environ Toxicol Chem.

[35] Rao GS (1970). Identity of peyocactin, an antibiotic from peyote (Lophophora williamsii), and hordenine. J Pharm Pharmacol.

[36] Ren Y, Deng J, Huang J, Wu Z, Yi L, Bi Y, Chen F (2021). Using green alga *Haematococcus pluvialis* for astaxanthin and lipid co-production: advances and outlook. Biores Technol.

[37] Rodil R, Quintana JB, Cela R (2012). Oxidation of synthetic phenolic antioxidants during water chlorination. J Hazard Mater.

[38] Rychen G, Aquilina G, Azimonti G, Bampidis V, Bastos ML, Bories G, Chesson A, Cocconcelli PS, Flachowsky G, Kolar B, Kouba M, López-Alonso M, Puente SL, Mantovani A, Mayo B, Ramos F, Saarela M, Villa RE, Wallace RJ, Wester P, Lundebye A-K, Nebbia C, Renshaw D, Innocenti MLJ (2018). Scientific Opinion on the safety and efficacy of butylated hydroxyanisole (BHA) as a feed additive for all animal species. EFSA J.

[39] Ryu K (2010). The characteristics of performance and exhaust emissions of a diesel engine using a biodiesel with antioxidants. Biores Technol.

[40] Sahraee S, Milani JM, Regenstein JM, Kafil HS (2019). Protection of foods against oxidative deterioration using edible films and coatings: a review. Food Biosci.

[41] Santana CS, Aguiar A (2015). Effect of biological mediator, 3-hydroxyanthranilic acid, in dye decolorization by Fenton processes. Int Biodeterior Biodegradation.

[42] Santana CS, Nicodemos Ramos MD, Vieira Velloso CC, Aguiar A (2019). Kinetic evaluation of dye decolorization by fenton processes in the presence of 3-hydroxyanthranilic acid. Int J Environ Res Public Health.

[43] Shah MMR, Liang Y, Cheng JJ, Daroch M (2016). Astaxanthin-producing green microalga *Haematococcus pluvialis*: From single cell to high value commercial products. Front Plant Sci.

[44] Specht E, Miyake-Stoner S, Mayfield S (2010). Micro-algae come of age as a platform for recombinant protein production. Biotech Lett.

[45] Stanier RY, Kunisawa R, Mandel M, Cohen-Bazire G (1971). Purification and properties of unicellular blue-green algae (order Chroococcales). Bacteriol Rev.

[46] Strittmatter M, Guerra T, Silva J, Gachon CMM (2016). A new flagellated dispersion stage in *Paraphysoderma sedebokerense*, a pathogen of Haematococcus pluvialis. J Appl Phycol.

[47] Tai C, Peng J-F, Liu J-F, Jiang G-B, Zou H (2004). Determination of hydroxyl radicals in advanced oxidation processes with dimethyl sulfoxide trapping and liquid chromatography. Anal Chim Acta.

[48] Torres-Tiji Y, Fields FJ, Mayfield SP (2020). Microalgae as a future food source. Biotechnol Adv.

[49] Torres MA, Jones JDG, Dangl JL (2006). Reactive oxygen species signaling in response to pathogens. Plant Physiol.

[50] Waterhouse RM, Seppey M, Simão FA, Manni M, Ioannidis P, Klioutchnikov G, Kriventseva EV, Zdobnov EM (2017). BUSCO applications from quality assessments to gene prediction and phylogenomics. Mol Biol Evol.

[51] Wayama M, Ota S, Matsuura H, Nango N, Hirata A, Kawano S (2013). Three-dimensional ultrastructural study of oil and astaxanthin accumulation during encystment in the green alga *Haematococcus pluvialis*. PLoS ONE.

[52] Wellburn AR (1994). The spectral determination of chlorophylls a and b, as well as total carotenoids, using various solvents with spectrophotometers of different resolution. J Plant Physiol.

[53] Williams GM, Iatropoulos MJ, Whysner J (1999). Safety assessment of butylated hydroxyanisole and butylated hydroxytoluene as antioxidant food additives. Food Chem Toxicol.

[54] Xu X, Liu A, Hu S, Ares I, Martínez-Larrañaga M-R, Wang X, Martínez M, Anadón A, Martínez M-A (2021). Synthetic phenolic antioxidants: metabolism, hazards and mechanism of action. Food Chem.

[55] Zhang Y, Ma J, Zhou S, Ma F (2015). Concentration-dependent toxicity effect of SDBS on swimming behavior of freshwater fishes. Environ Toxicol Pharmacol.

[56] Zhou J-W, Luo H-Z, Jiang H, Jian T-K, Chen Z-Q, Jia A-Q (2018). Hordenine: a novel quorum sensing inhibitor and antibiofilm agent against *Pseudomonas aeruginosa*. J Agric Food Chem.

