# Supplementary material for: Oxidative stress facilitates infection of the unicellular alga Haematococcus pluvialis by the fungus Paraphysoderma sedebokerense
Source: Biotechnol Biofuels Bioprod. 2022 May 20;15:56. doi: 10.1186/s13068-022-02140-y (PMC9123766; doi:10.1186/s13068-022-02140-y)
Supplement: Supplementary file 1 — Additional file 1: Table S1. Activity of the filtrated SPI in degrading cellular carbohydrates and pigments. Figure S1. Pigment degrading activity of SPI harvesting from the algal cultures at various infection stage. Figure S2. Morphological changes of the algal cells treated with the screened metabolites. Figure S3. Fe3+ reducing activity of the screened metabolites. Figure S4. Hydroxyl radical production of the screened metabolites. [file 13068_2022_2140_MOESM1_ESM.docx]

**Table S1.** **The activity of the filtrated SPI in degrading cellular carbohydrates and pigments.** Control, algal cells grow on BG11 medium. SPI, algal cells grow on SPI. Filtrated SPI, algal cells grow on 3000 Da filtrated SPI. The quantitative data were presented as mean ± S.D. (n=3). **, p < 0.01 (Student’s t-test).

| Samples | Carbohydrates (%) | Pigments (%) |
| --- | --- | --- |
| Control | 100±9.32 | 100±2.52 |
| SPI | 68.78±1.58^**^ | 78.83±1.88^**^ |
| Filtrated SPI | 64.82±5.17^**^ | 78.95±0.09^**^ |


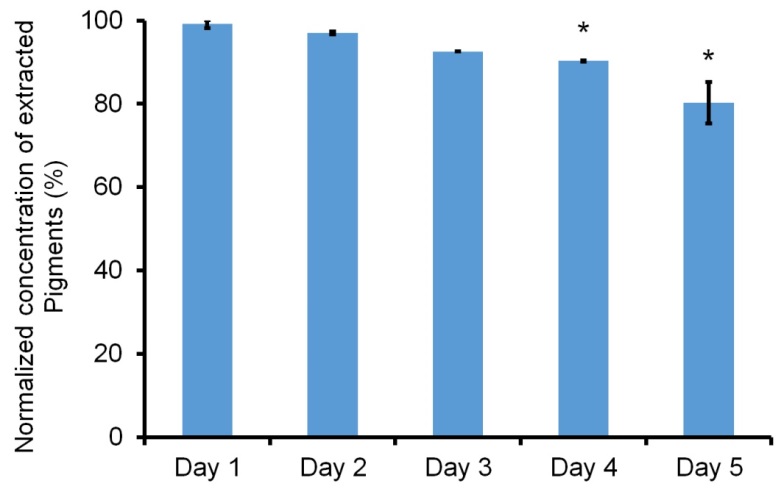


**Figure S1.** **The pigment degrading activity of SPI harvesting from the algal cultures at various infection stage.** The extracted pigments of each SPIs-treated algal sample were compared to control (algal cells treated with BG11 medium, as 100%). The quantitative data were presented as mean ± S.D. (n=3). *, *p* < 0.05 (Student’s t-test).


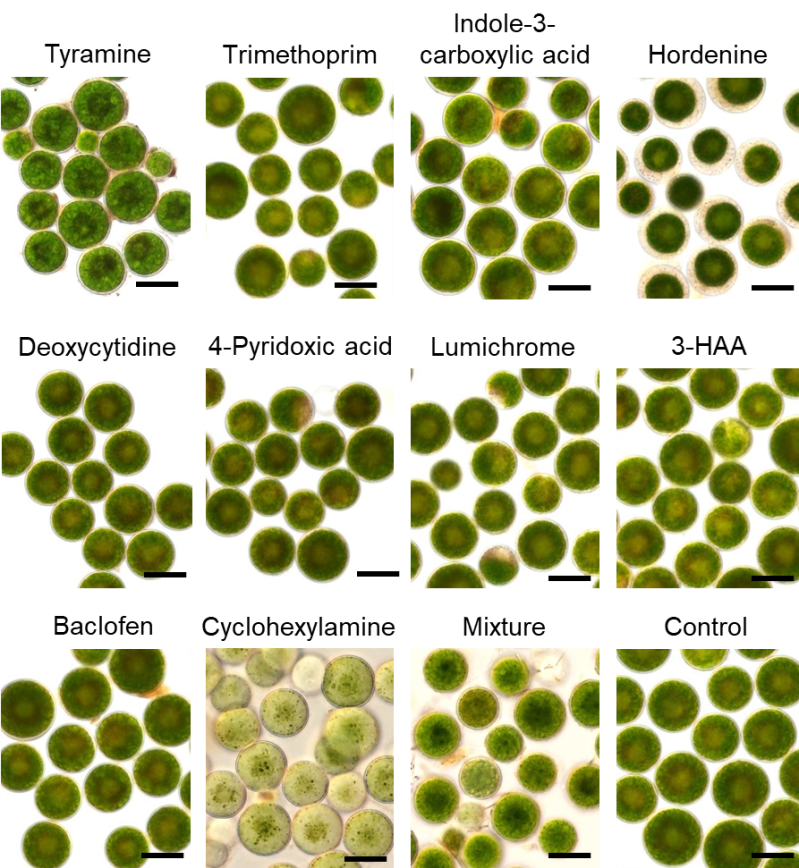


**Figure S2. Morphological changes of the algal cells treated with screened metabolites.** Tyramine and hordenine degraded the intracellular pigments without disruption of cell structure, whereas cyclohexylamine directly disrupted the cell structure and caused the effluxion of pigments, which was not resulted from degradation of pigments. Control sample was algal cells treated with BG11 medium, and the mixture sample was algal cells treated with 10 metabolites that mixed in equal volume. Bars=20μm.


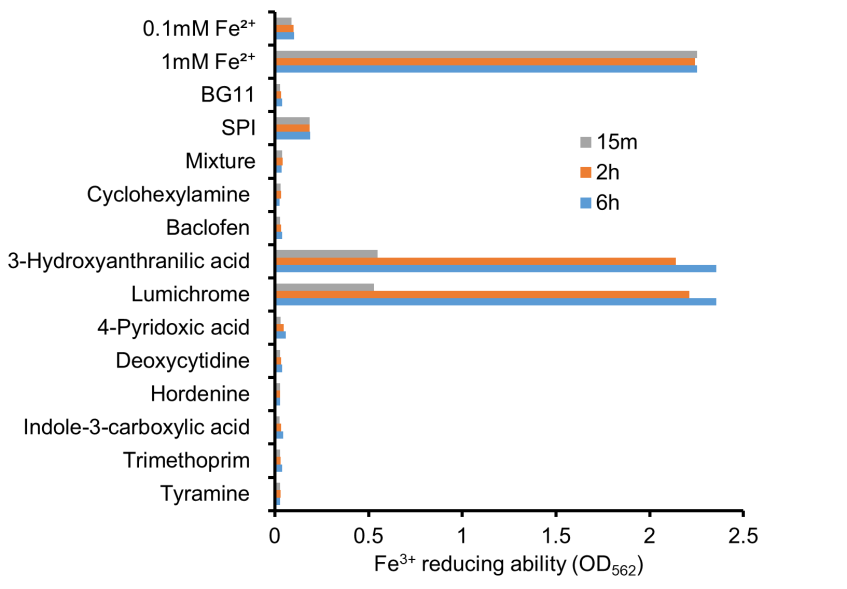


**Figure S3. Fe^3+^ reducing activity of the screened metabolites.** The mixture sample was algal cells treated with 10 reagents that mixed in equal volume.


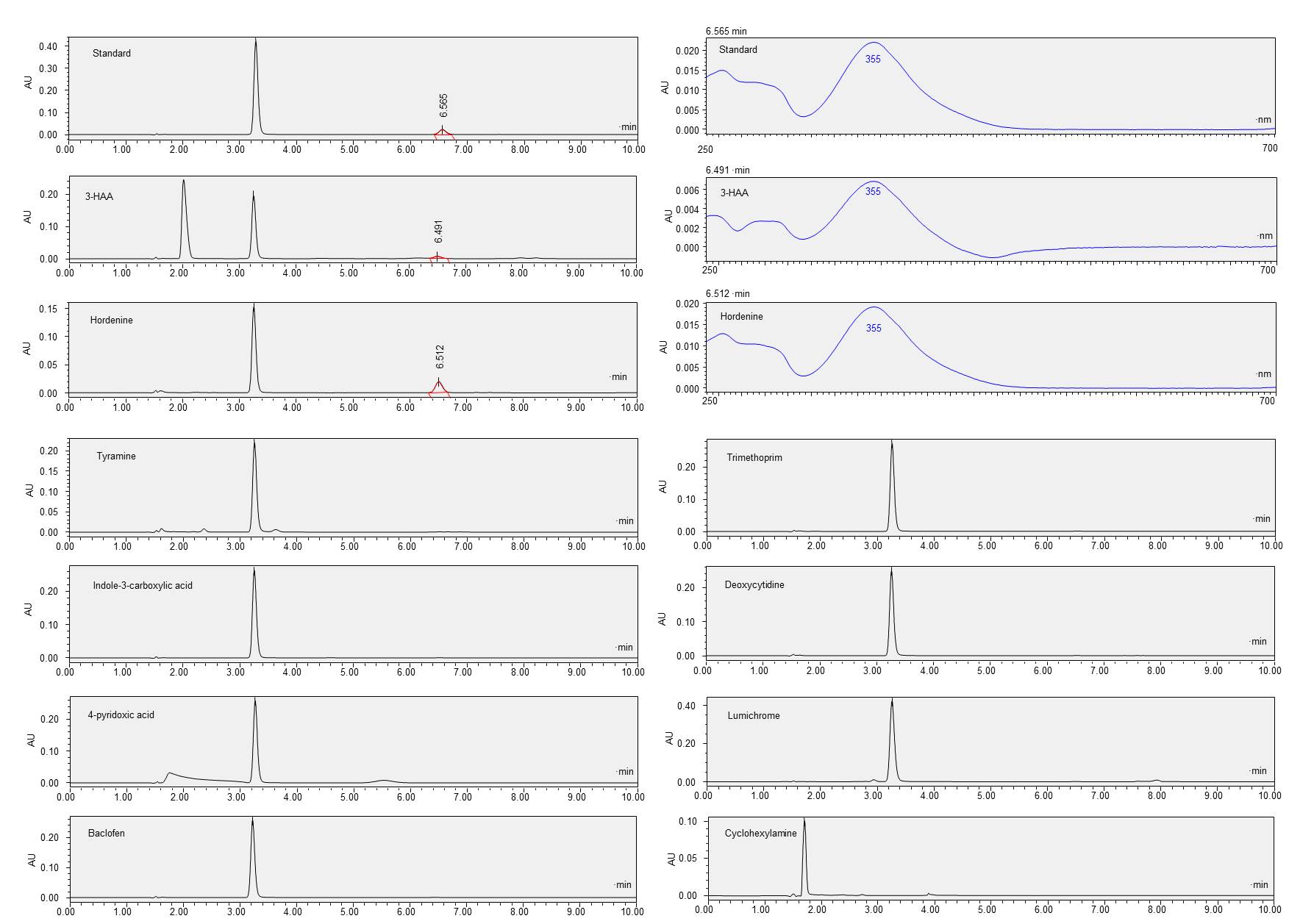


**Figure S4. Hydroxyl radical productivity of the screened metabolites.** 3-HAA and hordenine generated hydroxyl radical in the assay with DMSO as substrate, no hydroxyl radical productivity was detected with the other 8 metabolites by HPLC.
